# Supplementary material for: An electroluminescent and tunable cavity-enhanced carbon-nanotube-emitter in the telecom band
Source: Nat Commun. 2023 Jul 4;14:3933. doi: 10.1038/s41467-023-39622-y (PMC10319711; doi:10.1038/s41467-023-39622-y)
Supplement: Supplementary file 1 — Supplementary Information [file 41467_2023_39622_MOESM1_ESM.pdf]

Supplementary Information

An electroluminescent and tunable cavity-enhanced carbon-nanotube-emitter in the telecom band

Anna P. Ovvyan[1], Min-Ken Li[2,4], Helge Gehring[1], Fabian Beutel[1], Sandeep Kumar[3], Frank Hennrich[2], Li Wei[5], Yuan Chen[5], Felix Pyatkov[3,4], Ralph Krupke[2,3,4] and Wolfram H.P. Pernice[1,6,7,*]

[1]University of Münster, Physikalisches Institut, Center for Nanotechnology, Heisenbergstr. 11, 48149 Münster, Germany

[2]Institute of Quantum Materials and Technologies, Karlsruhe Institute of Technology, 76021 Karlsruhe, Germany

[3]Institute of Nanotechnology, Karlsruhe Institute of Technology, 76021 Karlsruhe, Germany

[4]Institute of Materials Science, Technische Universität Darmstadt, 64287 Darmstadt, Germany

[5]The University of Sydney, School of Chemical and Biomolecular Engineering, Darlington, NSW 2006, Australia

[6]Center for Soft Nanoscience, Busso-Peuss-Str. 11, 48149 Münster, Germany

[7]Kirchhoff-Institut for Physics, Im Neuenheimer Feld 227, 69120 Heidelberg, Germany

*Corresponding author: * wolfram.pernice@kip.uni-heidelberg.de*

Table of contents

1. **PhC cavity optimization**
2. **Influence of the electrode material on the Q-factor**
3. **Telecom NCG-based incandescent nanoemitter at room temperature**
4. **LDOS enhancement map of the cross-bar PhC cavity along the transverse direction**
5. **EL spectra of an sCNT coupled to a cross-bar PhC cavity at room temperature**
6. **Dynamic control of EL from a cavity-integrated sCNT**
7. **I-V curves of a sCNT and a NCG strip**
8. **Electron temperature of the NCG-based incandescent nanoemitter**
9. **Independence of the source-drain current of the NCG-based incandescent nanoemitter of the gate voltage**
10. **Hybrid NCG-Si_3N_4 PhC devices on-chip**

1. PhC cavity optimization

The cross-bar Photonic Crystal (PhC) cavity is optimized for the enhancement of electroluminescence (EL) emitted from the coupled sCNT (9,8) emitters. The PhC cavity contains two identical nonuniform Bragg mirrors, each mirror having *N* segments with a period *a* (Supplementary Fig. 1a). To ensure single mode fundamental transverse-electrical mode (TE0) guidance in the waveguide in the wavelength range of interest (1400−1470 nm), the waveguide width is set to 1450 nm, while the thickness is given by the thickness of silicon nitride layer (335 nm). The cavity holes in the mirror segments, characterized via the filling fraction of each segment $ff = \frac{\pi * l_1 l_2}{4 * w * a}$, are determined and further optimized by utilization of the open-source MPB software [1]. We numerically find that an elliptic shape of holes with the ratio of major axis $\frac{l_1}{l_2} = 3.7$ to the mirror axis ensures higher mirror strength and a broader bandgap in comparison with a round shape of holes, which at the end translates into a higher Q-factor of the PhC cavity.

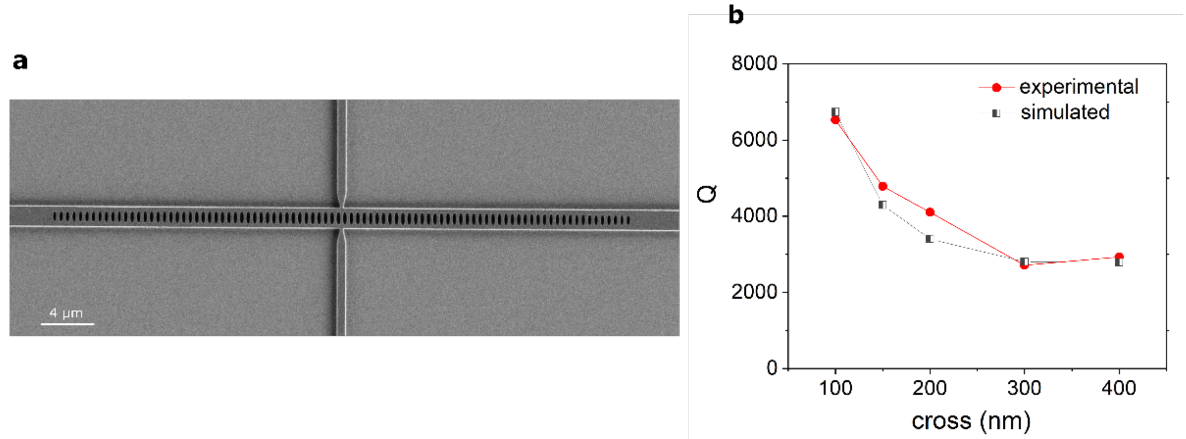

Supplementary Figure 1. a) *SEM image of a fabricated cross-bar PhC cavity.* ***b)*** *Experimentally measured (red points) and simulated (black curve) Q-factor of the Ist-order TE-like resonance mode for different widths of the crossed waveguide. Each Bragg mirror of the PhC cavity consists of N = 45 segments with periodicity a = 465 nm,* ff_f *=0.28,* ff_l *=0.16.*

A subsequent numerical optimization step for the cross-bar PhC cavity was performed through several cycles of 3D Finite Difference Time Domain (FDTD) simulations using the open-source MEEP software [2]. To provide smooth transition of resonance modes into waveguide modes we imposed quadratic interpolation of the filling fraction of the segments in

each Bragg mirror $ff(n) = ff_f - \frac{ff_f - ff_l}{(N-1)^2}(n-1)^2$, where we used ff_f – filling fraction of the first (inner) segment, ff_l – filling fraction of the last (outer) segment, n – count number of segment, N – total number of mirror segments (periods).

We characterize the optimized cross-bar PhC cavity via the quality factor (Q-factor), measured by transmission measurements utilizing a broadband excitation source (supercontinuum light source), coupled into and out of the cavity with 3D couplers. The simulated and experimentally measured Q-factors are in good agreement as shown in Supplementary Fig. 1b.

2. Influence of the electrode material on the Q-factor

To further illustrate the benefit of a cross-bar structure with nanocrystalline graphene electrodes atop, we analyze the effect of the electrode layer material on the odd resonance mode of the PhC cavity. Different widths of the cross-bars are equipped with nanocrystalline graphene (Supplementary Fig. 2a), gold electrodes (w_{gr}=280nm, L_{gr}=300nm) (Supplementary Fig. 2b), and without electrodes on top. The devices were characterized via transmission measurements. The experimentally determined Q-factor is shown in Supplementary Fig. 2c.

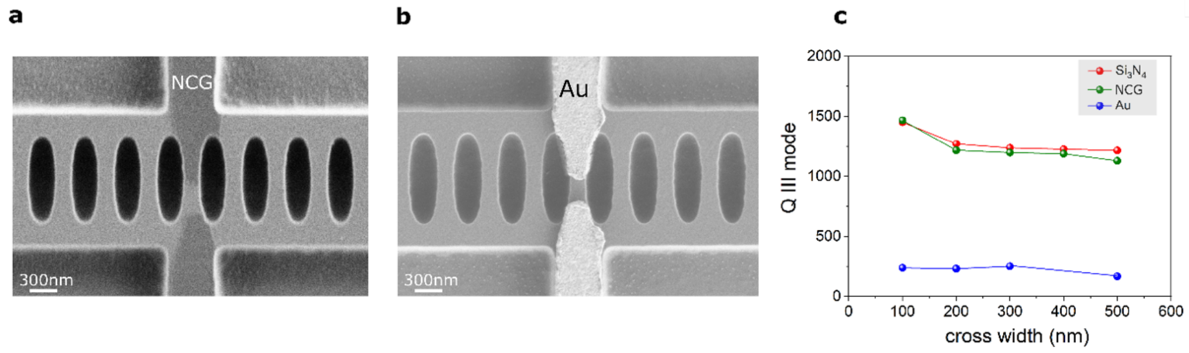

Supplementary Figure 2. Cross-bar PhC device equipped with nanocrystalline graphene nanoelectrodes. *SEM image of the cavity region of a cross-bar PhC device equipped with NCG* ***(a)*** *and gold* ***(b)*** *electrodes.* ***c)*** *Dependence of the Q-factor of the III-order resonance mode of a cross-bar PhC cavity on the width of cross-bar without electrodes (red curve), with NCG (green curve), and gold (blue curve) electrodes.*

We find that the effect of NCG electrodes on the Q-factor is very small, which can be seen by comparing the green and red curves, corresponding to similar cavities with nanocrystalline graphene electrodes and without deposited electrodes, respectively. In contrast,

gold electrodes significantly degrade the Q-factor of the III-order resonance mode by more than 4.9-7.8 times depending on the cross width, and suppress transmission of odd modes, since the antinode of the mode electric field lies in the center of the cavity. An increase of the width of the cross-bar w_{cr} leads to a decrease of Q-factor for all considered cases. We note that the measured Q-factor was limited by the resolution of the spectrometer as well as the coherence length of the employed supercontinuum light source.

3. Telecom NCG-based incandescent nanoemitter at room temperature

We characterize the cross-bar PhC cavity with a nanocrystalline graphene strip via transmission measurements. A broadband light source (supercontinuum laser) is coupled into the chip using the 3D coupler port A on the waveguide leading to the cavity and the transmitted signal is read-out at port C (Supplementary Fig. 3a).

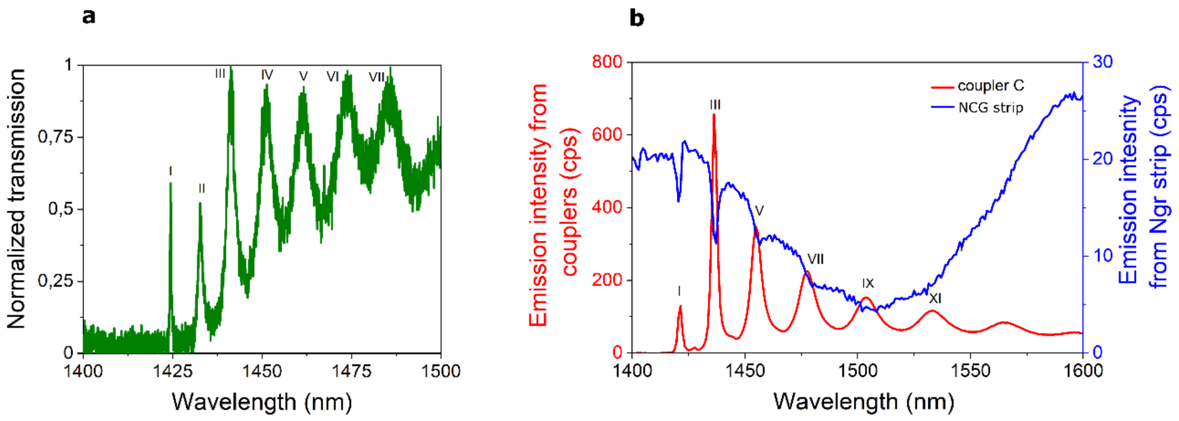

Supplementary Figure 3. NCG-based cavity-integrated light source. a) *Experimentally recorded transmission spectrum of a cross-bar PhC device with an integrated nanocrystalline graphene strip operating as an incandescent light source. The order of the cavity modes is labeled above the green curve.* ***b)*** *Spectra of enhanced incandescent emission extracted from coupler C (red curve) and the NCG strip (blue curve). The device is electrically excited with 120uA current and the emitted output is projected onto a polarization parallel to the TE mode (measured with polarizer parallel to TE mode of waveguide), acquired at 300K. The enhancement factors and coupling efficiency of incandescent emission into the I, III, and V resonance modes are* F_I*=16.3,* F_{III} *=112.7, and* F_V *=55.6,* β_I*=94.2%,* β_{III} *=99.1%, and* β_V *=98.2%, respectively.*

4. LDOS enhancement map of the cross-bar PhC cavity along the transverse direction

Light emitted from a CNT source located in the center of the cavity is coupled to odd resonance modes and enhanced. Moving the emitter away from the cavity center along the transverse direction leads to reduced enhancement of the resonance modes, as depicted in Supplementary Fig. 4a.

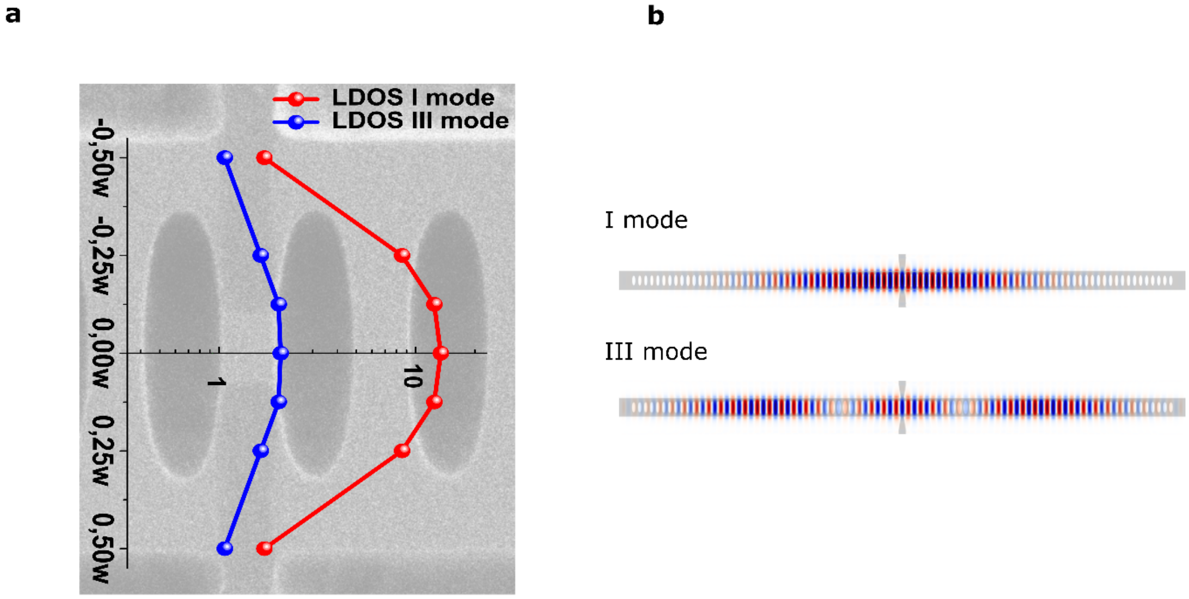

Supplementary Figure 4. Simulated LDOS enhancement spatial map and electric field distribution of resonance modes. a) *Simulated on-resonance LDOS enhancement spatial map of the EL from a sCNT sitting atop of the cavity region of a cross-bar PhC cavity equipped with NCG electrodes. The PhC consists of N = 25 holes in each Bragg mirror. The emitter position is fixed atop the cavity center and is varied along the lateral direction. The position of the source along the y-direction is normalized to the width of the cavity (w).* ***b)*** *Simulated electric filed distribution of the I-order and III-order resonance modes.*

5. EL spectra of sCNT coupled to a cross-bar PhC cavity at room temperature

We characterize the cross-bar PhC cavity before sCNT deposition via transmission measurements, where broadband light from a supercontinuum light source is coupled through 3D coupler port A to the cavity. The transmitted signal is read-out at port C (Supplementary Fig. 5a).

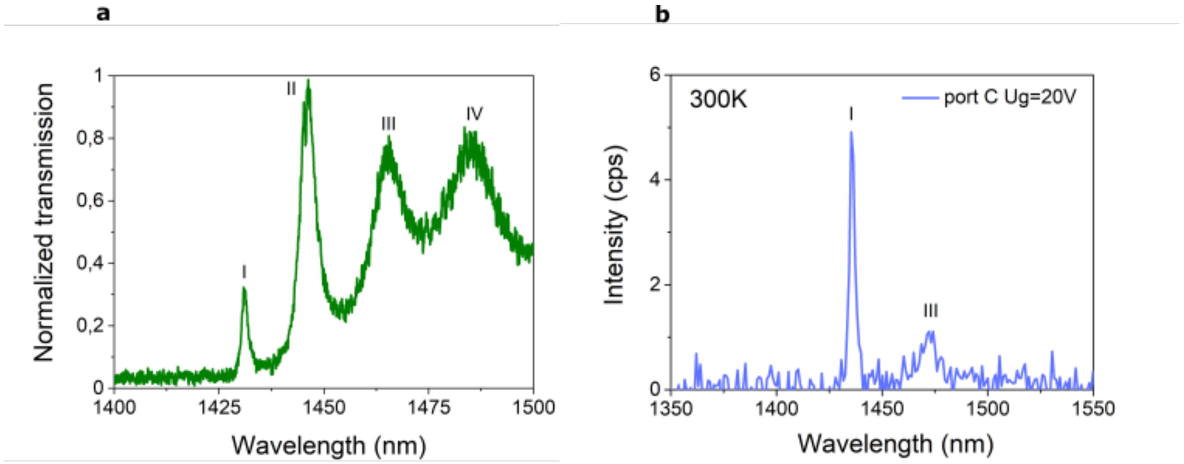

Supplementary Figure 5. Experimentally recorded spectra of hybrid NCG-Si_3N_4 PhC cavity. a) *Measured transmission spectrum of the hybrid device B1 with nanocrystalline graphene electrodes before sCNT integration.* ***b)*** *Spectra of enhanced EL outcoupled from 3D coupler port C of device 1 in the switched-on regime at U_g=20V, acquired at 300K.*

6. Dynamic control of EL from a cavity-integrated sCNT

As a proof of principle of our versatile approach of full electrical control of a single sCNT nanoemitter integrated in a custom-designed low-loss nanographene-photonic environment, we demonstrate in this section the measurements of another hybrid device, where the sCNT-EL is dynamically controlled by regulation of the back-gate voltage.

The electrically biased sCNT with simultaneously applied back-gate voltage (U_g= - 25V) emits enhanced excitonic EL emission into the odd resonance modes of the cavity in agreement with LDOS-spatial maps (Fig. 4a, Supplementary Fig. 4a). The measured spectrum of light outcoupled from one of the ends (coupler C in Fig. 1c) of the investigated hybrid device is shown in Supplementary Fig. 6a (red curve). The data is obtained when the sCNT is charge neutral, which corresponds to the EL *switched-on* state. Changing the gate voltage from -25V to +30V leads to decrease of the intensity of the excitonic EL emission (light and dark green curves in Supplementary Fig. 6a). Switching the gate voltage to U_g=+30V leads to complete suppression of the excitonic EL emission, corresponding to the EL *switched-off* state (blue curve in Supplementary Fig. 6a). Thus, we obtain dynamic control of the sCNT enhanced EL emission with close to 100% on-off ratio (depth) via active electrical operation of the back-gate voltage.

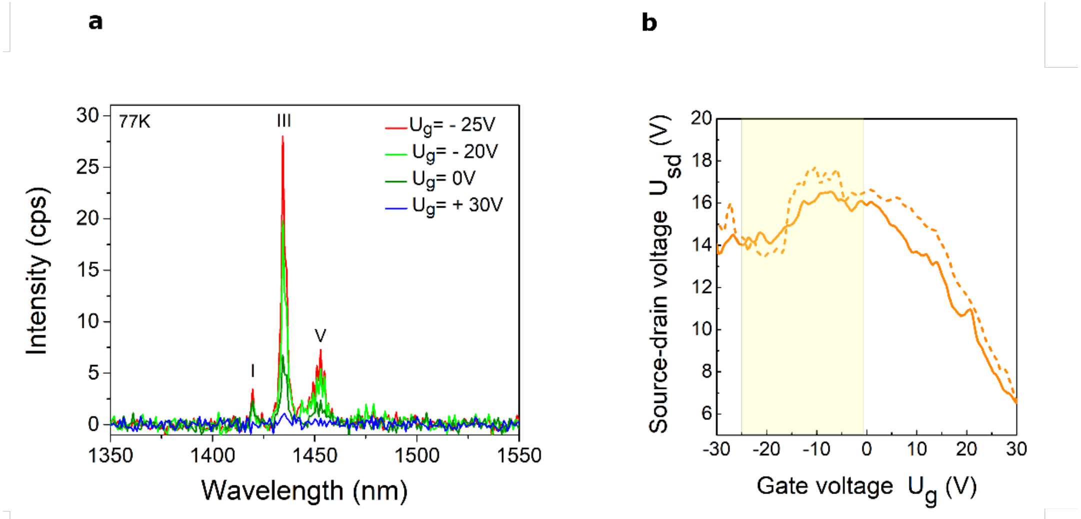

Supplementary Figure 6. Experimental dynamic control of EL from a cavity-integrated sCNT. a) *The spectra of EL coupled to odd resonance modes at 1419.5nm, 1434.4nm, 1452.9nm, acquired from coupler C of NCG-Si_3N_4 PhC cross-bar device gated with corresponding voltage U_g. At U_g=-25V – the EL is in the switched-on state (red curve) and at U_g =+30V – the EL is in the switched-off state (blue curve). The sCNT biasing current is constant I_{sd}=30nA. The cross-bar PhC cavity consists of N=45 segments in each Bragg mirror with a lattice period of a=457nm. The resonance modes are labelled.* ***b)*** *U_{sd}-U_g curve acquired at constant sCNT biasing current I_{sd}= 30nA. Forward and backward sweep traces are shown. The transport data shows negligible hysteresis between the forward and backward sweeps due to the cryogenic environment (77 K). The yellow area corresponds to the regime, in which the excitonic EL emission is in the switched-on state. All data is recorded at 77K.*

7. I-V curves of a sCNT and a NCG strip

I-V curves of a sCNT (9,8) integrated between NCG electrodes in the EL *switched-on* (blue curve) and EL *switched-off* (red curve) states are shown in Supplementary Fig. 7a. The I-V curve of the investigated nanocrystalline graphene strip with a narrow junction between NCG electrodes is demonstrated in Supplementary Fig. 7b. The linear increase of the source-drain current with applied voltage proves good Ohmic contact, indicating low resistance in case of the NCG strip (Supplementary Fig. 7b), while in the case of the sCNT the behavior of I-V curve

is non-linear (Supplementary Fig. 7a), which is a signature of a non-ohmic contact, implying a higher barrier between the polymer-wrapped (9,8) sCNT and the NCG nano-electrodes.

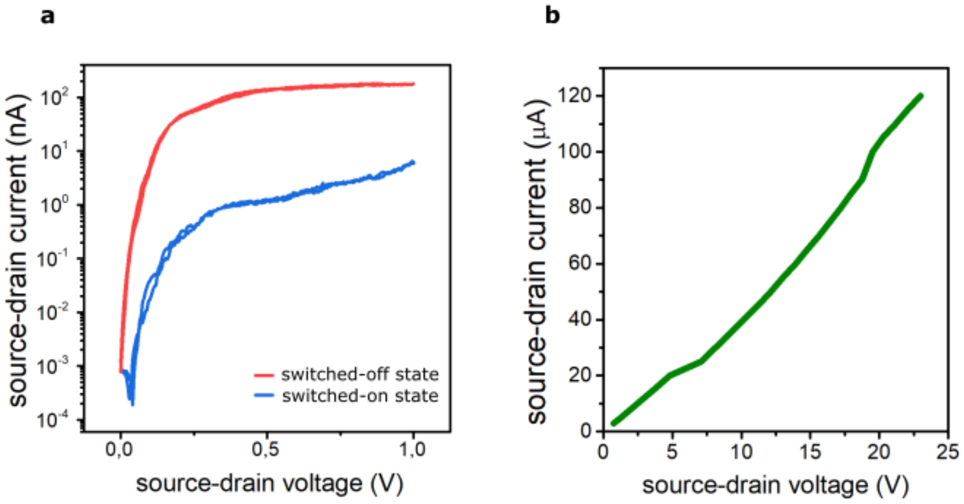

Supplementary Figure 7. Measured I-V curves of a sCNT (9,8) (a) and a NCG nano-strip (b). *The thickness of NCG is 5nm.*

8. Electron temperature of a NCG-based incandescent nanoemitter

We experimentally measured the free-space incandescent emission spectrum of an electrically biased nanocrystalline graphene strip placed on a Si_3N_4 waveguide (similar device design as shown in Fig. 3c, but NCG strip is placed on waveguide instead of cavity). The detected monotonic thermal spectrum (red curve) is shown in Supplementary Fig. 8.

Importantly, in our experiments (Fig. 3 and Supplementary Fig. 8), the utilized NCG strip (thickness 5 nm) consists of ~ 15 layers of single-layer graphene and is placed on a Si_3N_4 cavity or waveguide (the NCG strip is not suspended). These findings allow us to assume that NCG strip infrared emission results from an electronic temperature, since there are no significant non-equilibrium phonon distribution present [3] [4].

Thus, we fit the incandescent emission of the biased NCG strip at an applied electrical power of 2.87 mW (red curve in Supplementary Fig. 8a) with a grey-body theory (Planck's law, modified by an emissivity) (1), and determine the electron temperature of NCG $-$ T_e of ~ 1000K, as indicated by the dashed blue curve in Supplementary Fig. 8.

$$I(\lambda,T) = \varepsilon * \frac{2\pi hc^2}{\lambda^5} * \frac{1}{\exp\left(\frac{hc}{\lambda k_B T} - 1\right)}, \qquad (1)$$

Here we use $I(\lambda,T) -$ spectral energy density of thermal radiation from NCG strip, $h -$ Planck constant $6.626 * 10^{-34}$ $(J * sec)$, $k_B -$ Boltzmann const $1.380 * 10^{-23}$ $\left(\frac{J}{K}\right)$, $T -$ electron temperature (K) of NCG strip, $\lambda -$ wavelength of the emitted photons, $c -$ speed of light, ε – emissivity of NCG strip ($\varepsilon \approx 0.25$) [4] [5].

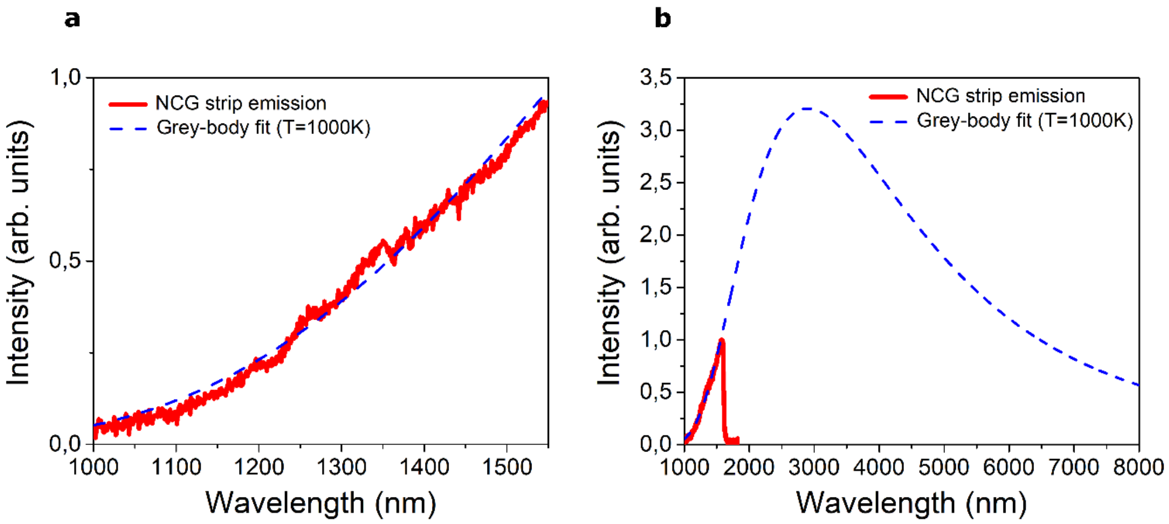

Supplementary Figure 8. Emission spectrum of a NCG strip nano-emitter on a Si_3N_4 waveguide. a) Recorded free-space thermal emission spectrum of an electrically biased NCG strip using 70 uA (source-drain voltage 41V). The spectrum from the NCG strip was acquired with the polarizer parallel to the TE mode of the waveguide. The waist of the NCG strip is 250 nm in width, thickness – 5 nm. Drop of the detected intensity at λ=1600nm is due to the cut-off of sensitivity of the utilized InGaAs photodiode linear array. Blue dashed curve: grey-body fit to the spectrum at the extracted temperature T=1000K according to equation (1), where the fully fitted spectrum is shown in **b)**.

9. Independence of the source-drain current of the NCG-based incandescent nanoemitter of gate voltage

The hybrid device with a NCG strip incorporated into the cavity region between NCG electrodes (Fig. 3a,c) is a thermal nano-source which allows us to probe the LDOS factor and provides optimal coupling of emitted light into the cavity modes at cryogenic and room temperature, as shown in Fig. 3 and Supplementary Fig. 3. We note that the supplied electric energy to the NCG strip is transformed into Joule heat and dissipated in the NCG, which emits incandescent emission. Thus, the electrical excitation of the thermal NCG strip nanoemitter (biasing current ~ hundreds of microamperes) is three-four orders of magnitude higher in comparison to the electroluminescent sCNT due to the incandescent nature of the NCG emission. To prove the incandescent nature of the emitted light we confirm independence of the source-drain current of the NCG on the change of gate voltage, which is shown in Supplementary Fig. 9.

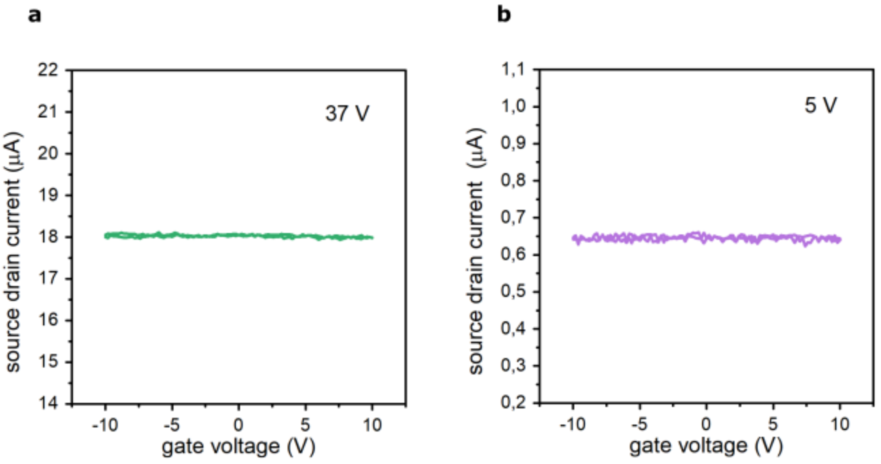

Supplementary Figure 9. Measured independence of the source-drain current of the NCG strip of the gate voltage. *The NCG biasing voltage is 37V (**a**) and 5V(**b**).*

10. Hybrid NCG-Si_3N_4 PhC devices on-chip

We detected successful coupling of sCNT EL emission into resonance modes in all hybrid NCG-PhC cavity devices which we have measured. The tailor-made cross-bar PhC cavities on the chip (Supplementary Fig. 10) contain various periods (a=453nm-466nm) and number of hole segments (N=25-45), which ensured variance of the resonance modes in a wide spectral range from 1400nm-1500nm in order to match the (9,8)-sCNT (central wavelength 1440 nm) emission line in the telecommunication band [6]. Our effective fabrication yield of NCG-Si_3N_4 devices is close to 95%. Electric field-assisted dielectrophoresis provides a stable deposition method for sCNTs with the effective yield of 83% in our case. Our optimized full fabrication protocol enables reproducibility for photonic applications in the telecommunication band.

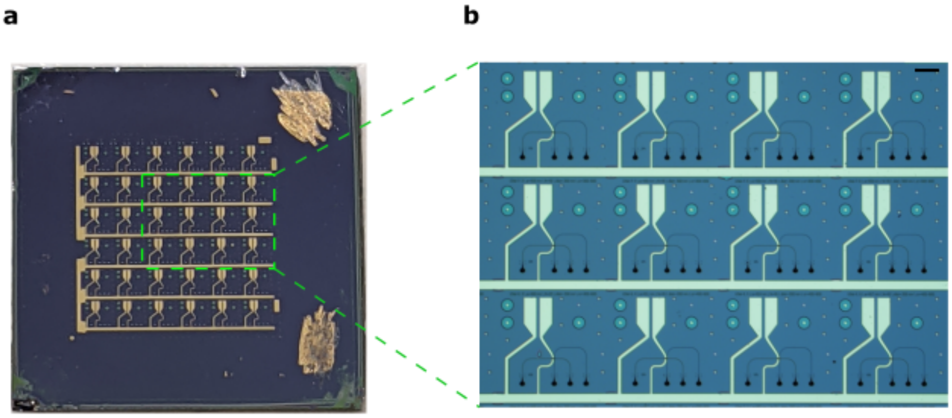

Supplementary Figure 10. Fabricated hybrid devices on-chip. a) *Photograph of the chip (1cm^2) for full dynamic electrical control of enhanced sCNT electroluminescent emission via operation of the back-gate voltage. The chip area is 5.4mm^2.* ***b)*** *Optical micrograph of a NCG-Si_3N_4 PhC cavity devices equipped with 3D couplers. Scale-bar: 200 µm.*

Supplementary References

[1] S. G. Johnson and J. D. Joannopoulos. Block-iterative frequency-domain methods for Maxwell's equations in a planewave basis. *Optics Express* **8** (2001).

[2] A. F. Oskooi, D. Roundy, M. Ibanescu, P. Bermel, J. D. Joannopoulos, S. G. Johnson. MEEP: A Flexible Free-Software Package for Electromagnetic Simulations by the FDTD Method. *Computer Physics Communications* **181** (2010).

[3] M. Freitag, H-Y. Chiu, M. Steiner, V. Perebeinos, P. Avouris. Thermal infrared emission from biased graphene. *Nature Nanotech* **5**, 497–501 (2010).

[4] Y. Kim, H. Kim, Y. Cho, *et al.* Bright visible light emission from graphene. *Nature Nanotech* **10**, 676–681 (2015).

[5] S-E Zhu, S. Yuan, G. C. A. M. Janssen. Optical transmittance of multilayer graphene. *EPL* **108** 17007 (2014).

[6] M. Gaulke, A. Janissek, N. A. Peyyety, I. Alamgir, A. Riaz, S. Dehm, H. Li, U. Lemmer, B. S. Flavel, M. M. Kappes, F. Hennrich, L. Wei, Y. Chen, F. Pyatkov, and R. Krupke. Low-Temperature Electroluminescence Excitation Mapping of Excitons and Trions in Short-Channel Monochiral Carbon Nanotube Devices. *ACS Nano* **14,** 2709–2717 (2020).
